# Ajustando a RFR por Preditores de Discordância, “A RFR Ajustada”: Uma Metodologia Alternativa para Melhorar a Capacidade Diagnóstica dos Índices Coronarianos

**DOI:** 10.36660/abc.20220176

**Published:** 2022-11-01

**Authors:** Diego Fernández-Rodríguez, Juan Casanova-Sandoval, Ignacio Barriuso, Kristian Rivera, Imanol Otaegui, Bruno García del Blanco, Teresa Gil Jiménez, Manuel López-Pérez, Marcos Rodríguez-Esteban, Francisco Torres-Saura, Víctor Jiménez Díaz, Raymundo Ocaranza-Sánchez, Vicente Peral Disdier, Guillermo Sánchez Elvira, Fernando Worner

**Affiliations:** 1 Hospital Universitari Arnau de Vilanova Lleida Espanha Hospital Universitari Arnau de Vilanova, Lleida – Espanha; 2 Institut de Recerca Biomédica de Lleida Lleida Espanha Institut de Recerca Biomédica de Lleida (IRBLleida), Lleida – Espanha; 3 Hospital Universitari Vall d´Hebron Barcelona Espanha Hospital Universitari Vall d´Hebron, Barcelona – Espanha; 4 Hospital Universitario Clínico San Cecilio Granada Espanha Hospital Universitario Clínico San Cecilio, Granada – Espanha; 5 Hospital Universitario Nuestra Señora de Candelaria Tenerife Espanha Hospital Universitario Nuestra Señora de Candelaria, Tenerife – Espanha; 6 Hospital Universitario de Vinalopó Elche Espanha Hospital Universitario de Vinalopó, Elche – Espanha; 7 Hospital Universitario Alvaro Cunqueiro Vigo Espanha Hospital Universitario Alvaro Cunqueiro, Vigo – Espanha; 8 Hospital Universitario Lucus Augusti Lugo Espanha Hospital Universitario Lucus Augusti, Lugo – Espanha; 9 Hospital Son Espases Palma de Mallorca Espanha Hospital Son Espases, Palma de Mallorca – Espanha; 10 Complejo Hospitalario de Navarra Pamplona Espanha Complejo Hospitalario de Navarra, Pamplona – Espanha

**Keywords:** Angina, Reserva de Fluxo Fracionado, Relação do Ciclo Completo de Repouso, Sensibilidade, Especificidade

## Abstract

**Fundamento:**

Os limiares de corte para a “relação do ciclo completo de repouso” (RFR) oscilam em diferentes séries, sugerindo que as características da população podem influenciá-los. Da mesma forma, foram documentados preditores de discordância entre a RFR e a reserva de fluxo fracionado (FFR). O Estudo RECOPA, mostrou que a capacidade diagnóstica está reduzida na “zona cinzenta” da RFR, tornando necessária a realização de FFR para descartar ou confirmar isquemia.

**Objetivos:**

Determinar os preditores de discordância, integrar as informações que eles fornecem em um índice clínico-fisiológico: a “RFR Ajustada”, e comparar sua concordância com o FFR.

**Métodos:**

Usando dados do Estudo RECOPA, os preditores de discordância em relação à FFR foram determinados na “zona cinzenta” da RFR (0,86 a 0,92) para construir um índice (“RFR Ajustada”) que pesaria a RFR juntamente com os preditores de discordância e avaliar sua concordância com a FFR.

**Resultados:**

Foram avaliadas 156 lesões em 141 pacientes. Os preditores de discordância foram: doença renal crônica, cardiopatia isquêmica prévia, lesões não envolvendo a artéria descendente anterior esquerda e síndrome coronariana aguda. Embora limitada, a “RFR Ajustada” melhorou a capacidade diagnóstica em comparação com a RFR na “zona cinzenta” (AUC-RFR = 0,651 versus AUC-“RFR Ajustada” = 0,749), mostrando também uma melhora em todos os índices diagnósticos quando foram estabelecidos limiares de corte otimizados (sensibilidade: 59% a 68%; especificidade: 62% a 75%; acurácia diagnóstica: 60% a 71%; razão de verossimilhança positiva: 1,51 a 2,34; razão de verossimilhança negativa: 0,64 a 0,37).

**Conclusões:**

Ajustar a RFR integrando as informações fornecidas pelos preditores de discordância para obter a “RFR Ajustada” melhorou a capacidade diagnóstica em nossa população. Mais estudos são necessários para avaliar se os índices clínico-fisiológicos melhoram a capacidade diagnóstica da RFR ou de outros índices coronarianos.

## Introdução

Os índices da fisiologia coronária são uma ferramenta essencial na tomada de decisão relacionados a pacientes com doença isquêmica do coração.^1,2^ Na prática clínica, são utilizados de forma dicotômica para determinar o significado funcional das lesões coronárias.^3,4^ No entanto, a escolha dos limiares de corte usando a análise da curva característica de operação do receptor (ROC) significa que alterações mínimas no limiar de corte podem levar a alterações relevantes na sensibilidade e especificidade.^5–7^ Além disso, os limiares de corte otimizados variam entre séries, sugerindo que a heterogeneidade das populações estudadas pode influenciar a capacidade diagnóstica desses índices.^5–7^

O conceito de “zona cinzenta” na reserva de fluxo coronariano ou reserva de fluxo fracionado (FFR)^8,9^ refere-se a uma faixa de valores próximos ao limiar de corte cujos extremos têm altos valores preditivos para confirmar ou descartar isquemia. Esse conceito também tem sido estudado com índices de repouso não hiperêmicos.^10,11^ O estudo de Casanova-Sandoval et al.^11^ foi um estudo de validação da “relação do ciclo completo de repouso” (RFR) contra a FFR na “vida real” que também avaliou a utilidade de uma estratégia híbrida de RFR e FFR para a avaliação funcional de estenoses na “zona cinzenta”.^11^

Por outro lado, existe um interesse crescente em determinar os preditores de discordância entre índices de repouso não hiperêmicos e FFR. Estudos recentes^12,13^ identificaram alguns deles para a RFR. No entanto, até onde sabemos, as informações desses preditores não têm sido utilizadas para melhorar a capacidade diagnóstica dos índices coronarianos em geral ou dos índices de repouso não hiperêmicos em particular.

Portanto, o objetivo do presente estudo foi o de determinar preditores de discordância entre RFR e FFR e integrar essas informações para construir um índice modificado de RFR, a “RFR Ajustada”, que permite melhorar a capacidade diagnóstica em relação à RFR na “zona cinzenta”.

## Materiais e métodos

### População do estudo

A população deste estudo foi selecionada usando dados do Casanova-Sandoval et al., cujos detalhes e resultados já foram publicados.^11^ Este estudo foi aprovado pelo Comitê de Ética de cada local, atendendo aos requisitos e às normas da Declaração de Helsinque e suas alterações posteriores, bem como aos regulamentos de proteção de dados aplicáveis.

Resumidamente, o estudo de Casanova-Sandoval et al.^11^ foi um estudo de validação de RFR versus FFR na prática padrão, onde 380 lesões coronárias em 311 pacientes foram avaliadas funcionalmente por pressão, obtendo valores de RFR e FFR. Os limiares para detecção de isquemia foram RFR ≤ 0,89 e FFR ≤ 0,80, com níveis de correlação (R^2^ = 0,81; p < 0,001), sensibilidade (76%) e especificidade (80%), semelhantes aos relatados por outros estudos da “vida real”. Porém, sua aplicação na população do estudo apresentou valores preditivos limitados (valor preditivo positivo [VPP] = 68%; valor preditivo negativo [VPN] = 80%). Assim, foi determinada uma “zona cinzenta” (RFR de 0,86 a 0,92) para avaliar o impacto funcional das estenoses usando ambas as técnicas (estratégia híbrida RFR-FFR), possibilitando a obtenção de altos valores preditivos (VPP = 91%; VPN = 92%) e a redução da administração de vasodilatadores em 58%.

Uma vez que os valores extremos da RFR permitem obter uma concordância muito alta, concentrando a discrepância entre ambas as técnicas na “zona cinzenta”, foram selecionadas apenas lesões com RFR de 0,86 a 0,92, incluindo um total de 156 lesões, correspondendo a 141 pacientes.

Determinação dos Preditores de Discordância e Estabelecimento da “RFR Ajustada”

A RFR^14^ é um índice de repouso não hiperêmico que avalia a significância hemodinâmica das estenoses coronarianas, identificando a relação mínima entre a pressão coronária distal à estenose (Pd) e a pressão aórtica (Pa) ao longo do ciclo cardíaco. O limiar de corte de RFR ≤ 0,89 é considerado o mais adequado para determinar a presença de isquemia, apesar das variações nos limiares de corte otimizados relatadas nas diferentes séries^11–17^. Na tentativa de afinar a concordância com a FFR na “zona cinzenta” (RFR de 0,86 a 0,92),^11^ foi realizada uma análise para determinar os preditores de discordância entre as duas técnicas, e as informações fornecidas por elas foram então incluídas na construção de um novo índice: a “RFR Ajustada”.

Primeiro, as lesões foram agrupadas em 4 grupos de acordo com o resultado do estudo funcional: RFR-/FFR- (verdadeiro negativo), RFR+/FFR- (falso positivo [FP]), RFR-/FFR+ (falso negativo [FN]) e RFR+/FFR+ (verdadeiro positivo), comparando as características clínicas e angiográficas entre cada grupo. Posteriormente, foram selecionados os grupos com resultados discordantes: RFR+/FFR- (FP) e RFR-/FFR+ (FN); preditores independentes de discordância foram então determinados para cada grupo. Por fim, foi construída a “RFR Ajustada”, incluindo a RFR e os preditores de discordância, atribuindo-lhes seus correspondentes coeficientes de ponderação.

### Análise estatística

Foram realizadas as análises estatísticas no software SPSS versão 20.0 (IBM Corp., Armonk, NY, EUA), com valores bicaudais de p < 0,05 considerados estatisticamente significativos. As variáveis categóricas foram apresentadas como números e frequências relativas (porcentagens), e as variáveis contínuas como média (desvio padrão) ou mediana com intervalo ou intervalo interquartil dependendo de sua distribuição. As variáveis contínuas foram comparadas pelo teste t de Student para amostras não pareadas e as variáveis categóricas foram comparadas pelo teste qui-quadrado ou teste exato de Fisher, conforme apropriado. Foram usados os testes U de Mann–Whitney para dados não paramétricos.

Para identificar preditores de discordância, tanto para FP quanto para FN, foram utilizados modelos de regressão logística binária, incluindo na análise multivariada final aqueles preditores com valores ≤ 0,10 na análise univariada. Os resultados foram dados como odds ratio (OR) com intervalo de confiança de 95% (IC 95%). Uma vez obtidos os preditores de discordância, foi construída a “RFR Ajustada” por meio de regressão linear para estabelecer um modelo preditivo de FFR que contempla o valor da RFR e os preditores de discordância, atribuindo-lhes um coeficiente que ponderou sua relevância usando o seguinte algoritmo:


“RFR Ajustada”: $$\text{p}(\text{y}=\text{FFR})=\text{RFR Ajustada}={\text{β}}_{\text{cte}}+{\text{β}}_{\text{RFR}}*\text{RFR}+\ldots \ldots +{\text{β}}_{\text{n}}*{\text{X}}_{\text{n}}$$

* Os coeficientes ponderados (β_i_) podiam ser positivos ou negativos, dependendo se os preditores eram fatores protetores ou de risco para ser FN ou FP.

Por fim, foram realizadas análises de sensibilidade e especificidade, estimando-se também o limiar de corte otimizado da “RFR Ajustada” para obter um valor de FFR ≤ 0,80, usando a análise da curva ROC. Razões de verossimilhança positiva e negativa (LR+ e LR-) também foram calculadas para RFR e “RFR Ajustada”, considerando a utilidade do teste da maneira seguinte:^14^

– LR+: < 2 (não útil); 2 a 5 (moderado); 5 a 10 (bom); > 10 (excelente).
– LR−: > 0,5 (não útil); 0,5 a 0,2 (moderado); 0,2 a 0,1 (bom); < 0,1 (excelente).

## Resultados

O presente estudo incluiu 141 pacientes, com um total de 156 lesões. Uma única lesão foi investigada na maioria dos pacientes, sendo 4 o número máximo de lesões avaliadas em 1 paciente.

### Características clínicas e angiográficas

As características basais por paciente são apresentadas na Tabela 1. A Tabela 2 mostra as características basais por lesão dos 4 grupos de comparação, observando-se que os FPs (RFR+/FFR-) tinham idade mais avançada, com maior prevalência de doença renal crônica e maior porcentagem de cardiopatia isquêmica crônica prévia. Em relação aos FNs (RFR-/FFR+), foi encontrada maior prevalência de tabagismo ativo e síndrome coronariana aguda. A Tabela 3 também mostra, por lesão, as características angiográficas e fisiológicas dos grupos de comparação, observando que os FNs (RFR-/FFR+) apresentaram maior porcentagem de lesões não envolvendo a artéria descendente anterior esquerda em comparação a todos os outros grupos. O segmento coronariano especificamente afetado é apresentado no Material Suplementar. Além disso, foi observado um gradiente nos valores de RFR e de Pd/Pa entre os 4 braços de comparação.

**Tabela 1:** Características basais por paciente

		Pacientes (n=141)
**Idade**, *(anos), média (DP)*		65,82 (12,3)
**Sexo feminino,** *n (%)*		39 (27,7%)
**IMC,** *(kg/m^2^)*, *média (DP)*		28,0 (4,8%)
**Hipertensão,** *n (%)*		104 (73,8%)
**Dislipidemia,** *n (%)*		93 (66%)
**Diabetes mellitus,** *n (%)*		50 (35,5%)
**Tabagismo atual,** *n (%)*		26 (18,4%)
**Cardiopatia isquêmica crônica prévia,** *n (%)*		40 (28,4%)
**Doença cerebrovascular,** *n (%)*		13 (9,2%)
**Fibrilação atrial,** *n (%)*		15 (10,6%)
**Doença vascular periférica,** *n (%)*		13 (9,2%)
**DPOC,** *n (%)*		9 (6,4%)
**Taxa de filtração glomerular,** *(mL/min/1,73 m^2^), média (DP)*		74,0 (31,2)
**Doença renal crônica,** *(taxa de filtração glomerular < 60 ml/min/1,73 m^2^), n (%)*		51 (36,7%)
**Indicação clínica**, *n (%)*		
	– Angina estável	102 (72,3%)
	– SCA sem supradesnivelamento do segmento ST: lesão culpada	21 (14,9%)
	– SCA sem supradesnivelamento do segmento ST: lesão não-culpada	10 (7,1%)
	– SCA com supradesnivelamento do segmento ST: lesão não-culpada	8 (5,7%)
**Lesões/paciente,** *(n), mediana (mínimo-máximo)*		1 (1-4)

DP: desvio padrão; DPOC: doença pulmonar obstrutiva crônica; IMC: índice de massa corporal; SCA: síndrome coronariana aguda.

**Tabela 2:** Características basais por lesão

		VN: RFR-/FFR- (n=60)	FP: RFR+/FFR- (n=41)	FN: RFR-/FFR+ (n=21)	VP: RFR+/FFR+ (n=34)	Valor p
**Idade**, *(anos), média (DP)*		63,0 (14,0)	71,5 (10,3)	62,1 (8,7)	66,2 (10,9)	**0,002**
**Sexo feminino,** *n (%)*		18 (30,0%)	13 (31,7%)	5 (23,8%)	4 (11,8%)	0,182
**IMC,** *(Kg/m2), média (DP)*		27,8 (4,6)	27,7 (4,8)	29,2 (5,5)	27,8 (4,2)	0,647
**Hipertensão,** *n (%)*		43 (71,7%)	30 (73,2%)	18 (85,7%)	24 (70,6%)	0,600
**Dislipidemia,** *n (%)*		38 (63,3%)	25 (61,0%)	13 (61,9%)	22 (64,7%)	0,989
**Diabetes mellitus,** *n (%)*		20 (33,3%)	18 (43,9%)	4 (19,0%)	14 (41,2%)	0,229
**Tabagismo atual,** *n (%)*		14 (23,3%)	3 (7,3%)	9 (42,9%)	7 (20,6%)	**0,013**
**Cardiopatia isquêmica crônica prévia,** *n (%)*		26 (43,3%)	5 (12,2%)	8 (38,1%)	7 (20,6%)	**0,004**
**Doença cerebrovascular,** *n (%)*		5 (8,3%)	5 (12,2%)	2 (9,5%)	2 (5,9%)	0,862
**Fibrilação atrial,** *n (%)*		4 (6,7%)	6 (14,6%)	1 (4,8%)	4 (11,8%)	0,468
**Doença vascular periférica,** *n (%)*		5 (8,3%)	4 (9,8%)	0 (0%)	6 (17,6%)	0,181
**DPOC,** *n (%)*		6 (10,0%)	0 (0%)	0 (0%)	4 (11,8%)	0,061
**Taxa de filtração glomerular,** (mL/min/1,73 m^2^), *média (DP)*		78,4 (33,0)	57,5 (26,0)	90,1 (20,4)	74,4 (29,5)	**< 0,001**
**Doença renal crônica,** *(taxa de filtração glomerular < 60 ml/min/1,73 m^2^), n (%)*		19 (31,7%)	26 (63,4%)	2 (9,5%)	9 (26,5%)	**< 0,001**
**Indicação clínica**, *n (%)*						**0,012**
	- Angina estável	46 (76,7%)	33 (80,5%)	9 (42,9%)	24 (70,6%)	
	- Síndrome coronariana aguda	14 (23,3%)	8 (19,5%)	12 (57,1%)	10 (29,4%)	

DP: desvio padrão; DPOC: doença pulmonar obstrutiva crônica; FFR: reserva de fluxo fracionado; FN: falso negativo; FP: falso positivo; IMC: índice de massa corporal; RFR: relação do ciclo completo de repouso; VN: verdadeiro negativo; VP: verdadeiro positivo.

**Tabela 3:** Características angiográficas e fisiológicas por lesão

		VN: RFR-/FFR- (n=60)	FP: RFR+/FFR- (n=41)	FN: RFR-/FFR+ (n=21)	VP: RFR+/FFR+ (n=34)	Valor p
**Administração de adenosina**, *n (%)*						0,343
	- Adenosina intravenosa	18 (30,0%)	17 (51,5%)	10 (47,6%)	10 (29,4%)	
	- Adenosina intracoronária	42 (70,0%)	24 (58,5%)	11 (52,4%)	24 (70,6%)	
**Tamanho do cateter guia**, *n (%)*						0,574
	- 5 French	2 (3,3%)	2 (4,9%)	0 (0%)	1 82,9%)	
	- 6 French	58 (96,7%)	39 (95,1%)	21 (100%)	32 (94,1%)	
	- 7 French	0 (0%)	0 (0%)	0 (0%)	1 (2,9%)	
**Vaso acometido**, *n (%)*						**0,019**
	- DAE	43 (71,7%)	33 (80,5%)	11 (52,4%)	30 (88,2%)	
	- Não DAE	17 (28,3%)	8 (19,5%)	10 (47,6%)	4 (11,8%)	
**Porcentagem de estenose**, *(%), média (DP)*		57 (11)	57 (10)	61 (10)	61 (9)	0,136
**Comprimento do vaso**, *n (%)*						0,716
	- <12 mm	30 (50,0%)	17 (41,5%)	11 (52,4%)	13 (38,2%)	
	- 12 a 25 mm	23 (38,3%)	20 (48,8%)	9 (42,9%)	14 (44,1%)	
	- >25 mm	7 (11,7%)	4 (9,8%)	1 (4,8%)	6 (17,6%)	
**Diâmetro do vaso**, *(mm), média (DP)*		3,01 (0,53)	2,89 (0,37)	2,93 (0,53)	2,92 (0,45)	0,636
**Índices coronarianos**, *média (DP)*						
	- RFR	0,91 (0,01)	0,88 (0,01)	0,91 (0,01)	0,87 (0,01)	**<0,001**
	- Pd/Pa	0,93 (0,02)	0,92 (0,02)	0,92 (0,02)	0,90 (0,03)	**<0,001**
	- FFR	0,86 (0,03)	0,85 (0,03)	0,76 (0,04)	0,76 (0,03)	**<0,001**

DAE: acometimento da artéria descendente anterior esquerda; DP: desvio padrão; FFR: reserva de fluxo fracionado; FN: falso negativo; FP: falso positivo; não DAE: não acometimento da artéria descendente anterior esquerda; Pd/Pa: relação entre a pressão coronária distal e a pressão aórtica; RFR: relação do ciclo completo de repouso; VN: verdadeiro negativo; VP: verdadeiro positivo.

### Determinação de preditores de discordância

A Tabela 4 mostra os preditores independentes de discordância para FP e FN. Em relação aos FPs (RFR+/FFR-), a doença renal crônica foi identificada como fator de risco independente para discordância (OR 3,224; 1,386 a 7,501; p = 0,007). Em contrapartida, a história de cardiopatia isquêmica crônica mostrou-se fator de proteção contra a discordância (OR 0,296; 0,102 a 0,858; p = 0,025). Em relação aos FNs (RFR-/FFR+), o contexto clínico de síndrome coronariana aguda (OR 3,687; 1,247 a 10,899; p = 0,018) e lesões em local diferente da artéria descendente anterior esquerda (OR 3,529; 1,231 a 10,118; p = 0,019) foram, por fim, identificados como fatores de risco independentes para discordância.

**Tabela 4:** Preditores independentes de discordância

RFR+/FFR- (falsos positivos)							
Análise univariada	OR	IC (95%)	Valor p	Análise multivariada	OR	IC (95%)	Valor p
**DRC**	**4,911**	**2,298-10,498**	**<0,001**	**DRC**	**3,224**	**1,386-7,501**	**0,007**
**Idade ≥ 75 anos**	**3,981**	**1,862-8,511**	**<0,001**	**CIC prévia**	**0,296**	**0,102-0,858**	**0,025**
**Lesões não DAE**	0,657	0,274-1,576	0,345				
**SCA**	0,532	0,224-1,266	0,150				
**CIC prévia**	**0,251**	**0,091-0,688**	**0,005**				
**Tabagismo atual**	**0,224**	**0,064-0,688**	**0,005**				
**RFR-/FFR+ (falsos negativos)**							
**Análise univariada**	**OR**	**IC (95%)**	**Valor p**	**Análise multivariada**	**OR**	**IC (95%)**	**Valor p**
**SCA**	**4,292**	**1,658-11,107**	**0,002**	**SCA**	**3,687**	**1,247-10,899**	**0,018**
**Tabagismo atual**	**3,469**	**1,314-9,154**	**0,009**	**Lesões não DAE**	**3,529**	**1,231-10,118**	**0,019**
**Lesões não DAE**	**3,323**	**1,285-8,594**	**0,010**				
**CIC prévia**	1,157	0,603-4,091	0,352				
**DRC**	**0,158**	**0,035-0,706**	**0,003**				
**Idade ≥ 75 anos**	**0,103**	**0,013-0,796**	**0,003**				

CIC: cardiopatia isquêmica crônica; DRC: doença renal crônica; FFR: reserva de fluxo fracionado; IC: intervalo de confiança; lesões não DAE: lesões que não acometem a artéria descendente anterior esquerda; OR: odds ratio; RFR: relação do ciclo completo de repouso; SCA: síndrome coronariana aguda.

### Geração da “RFR Ajustada”

Por fim, o valor da RFR e os preditores independentes foram incluídos no modelo para gerar a “RFR Ajustada”. O algoritmo com os coeficientes correspondentes a cada preditor é mostrado abaixo:


“RFR Ajustada”: $$\begin{array}{l} \text{RFR}\text{Ajustada}=0,009+0,912\text{*RFR}+0,023\text{*DRC}-0,019\text{*não}\text{DAE}\\ -0,017\text{*SCA}-\text{0,005*CIC prévia} \end{array}$$

* RFR: “relação do ciclo completo de repouso”; DRC: doença renal crônica (taxa de filtração glomerular < 60 ml/min/1,73 m^2^); não DAE: lesões que não acometem a artéria descendente anterior esquerda; SCA: síndrome coronariana aguda; CIC prévia: histórico de cardiopatia isquêmica crônica.

Este algoritmo mostra que a doença renal crônica (fator de risco para FP) é inserida com sinal positivo. As lesões que não acometem a artéria descendente anterior esquerda e a indicação de síndrome coronariana aguda (ambos fatores de risco para FN), bem como o histórico de cardiopatia isquêmica crônica (fator de proteção para FP), são inseridos com sinal negativo.

### Análise de sensibilidade e especificidade

**Figura 1:**
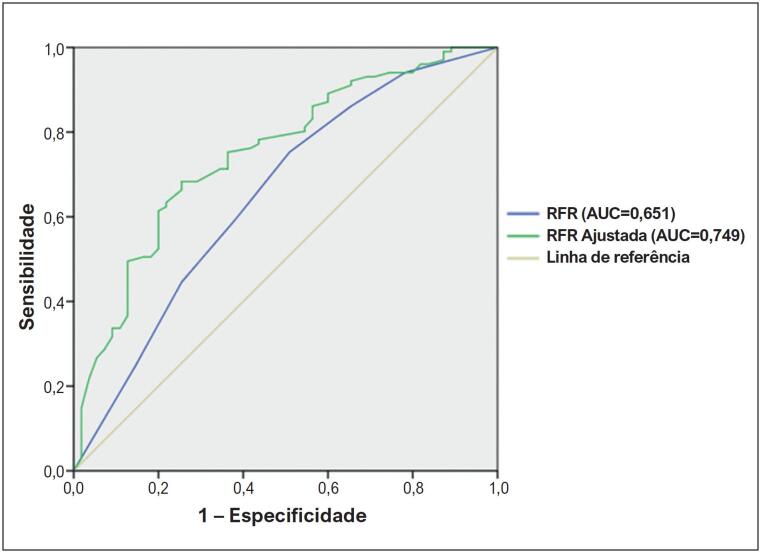
Curvas ROC de RFR versus FFR ≤ 0,80 e “RFR Ajustada” e FFR ≤ 0,80. AUC: área sob a curva; FFR: reserva de fluxo fracionado; ROC: característica de operação do receptor; RFR: relação do ciclo completo de repouso. A curva ROC mostrou uma AUC de 0,651 (0,559 a 0,744; p = 0,002) para a RFR, melhorando a AUC para a “RFR Ajustada” para 0,749 (0,669 a 0,828; p < 0,001) e estabelecendo um valor de 0,8172 como limite de corte otimizado para a “RFR Ajustada”.

**Figura 2:**
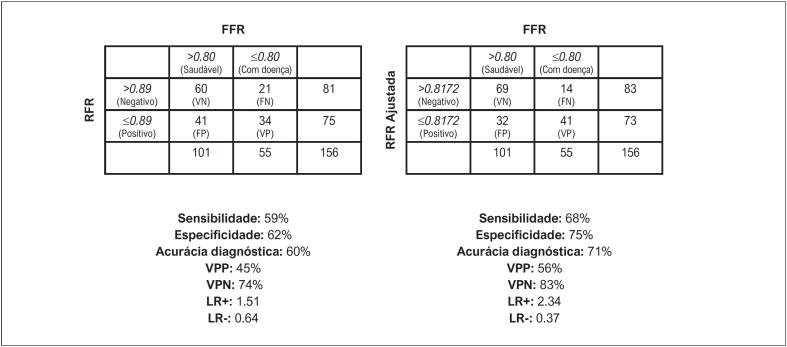
Comparação dos parâmetros diagnósticos de acordo com as Tabelas 2 × 2 para os limiares de corte de RFR (≤ 0,89) e “RFR Ajustada” (≤ 0,8172) versus FFR (≤ 0,80). FFR: reserva de fluxo fracionado; FN: falso negativo; FP: falso positivo; LR+: razão de verossimilhança positiva; LR-: razão de verossimilhança negativa; RFR: relação do ciclo completo de repouso; VN: verdadeiro negativo; VP: verdadeiro positivo; VPN: valor preditivo negativo; VPP: valor preditivo positivo.

A Figura 1 mostra as curvas ROC comparativas para a RFR e a “RFR Ajustada”. Observou-se aumento da área sob a curva (AUC) da “RFR Ajustada” em relação à RFR, de 0,651 para 0,749, determinando como limiar de corte otimizado uma “RFR Ajustada” de ≤ 0,8172 para detectar valores de FFR ≤ 0,80. Da mesma forma, a Figura 2 compara as tabelas de contingência de ambos os índices de acordo com os limiares de corte estabelecidos, com melhora na sensibilidade variando de 59% a 68%, especificidade variando de 62% a 75%, acurácia diagnóstica variando de 60% a 71%, VPP variando de 45% a 56% e VPN variando de 74% a 83%. De particular interesse é a melhora nas LRs, onde encontramos que, com o novo índice, LR+ aumentou de 1,51 para 2,34 e LR- diminuiu de 0,64 para 0,37. Desta maneira, a “RFR Ajustada” tem utilidade satisfatória em relação à RFR, que não é útil para discriminar pacientes na “zona cinzenta”.

## Discussão

Os principais achados do estudo foram: a) doença renal crônica, acometimento de outras artérias que não a descendente anterior esquerda, indicação de síndrome coronariana aguda e histórico de cardiopatia isquêmica crônica mostraram-se como preditores independentes de discordância na “zona cinzenta” da RFR em relação à FFR; e b) a modificação da RFR com a inclusão de preditores independentes de discordância (“RFR Ajustada”) possibilitou melhorar a capacidade diagnóstica do teste para os valores da “zona cinzenta”.

### Seleção da população-alvo: Por que a “zona cinzenta”?

Qualquer índice quantitativo contínuo utilizado de forma dicotômica envolve algum grau de incerteza diagnóstica em valores próximos ao limiar de corte estabelecido.^18^ O conceito de “zona cinzenta” para índices da fisiologia coronária surge de estudos de validação da FFR para a detecção de isquemia induzida por estenoses coronarianas epicárdicas.^8,9,19,20^ Este conceito foi subsequentemente estendido a outros índices de repouso não hiperêmicos como o *instantaneous wave-free ratio* e a RFR, mostrando que os valores extremos de índices de repouso não hiperêmicos apresentaram concordância muito alta com a FFR e, em valores próximos ao limiar de corte (“zona cinzenta”), a capacidade diagnóstica diminuiu.^10,11^ Visto que é plausível que os poucos resultados discordantes entre RFR e FFR em caso de valores extremos de RFR estejam relacionados principalmente a erros na técnica de medição, optou-se por restringir a determinação dos preditores de discordância à “zona cinzenta” da RFR.

Além disso, é relevante a proporção de pacientes avaliados por estudo fisiológico invasivo que se encontram na “zona cinzenta”, mostrando nos dados de RFR e *instantaneous wave-free ratio* que esta proporção de pacientes pode ultrapassar 40%.^10,11^ Portanto, consideramos essencial o desenvolvimento de ferramentas diagnósticas que possibilitem refinar o diagnóstico de índices de repouso não hiperêmicos e eventualmente outros índices fisiológicos invasivos, seja de circulação epicárdica ou microcirculação coronariana, para essa faixa de valores.

### Avaliação dos preditores de discordância: O território coronariano acometido prediz falsos positivos ou falsos negativos?

Considerando o tamanho limitado da amostra e a heterogeneidade das populações de estudo avaliadas pelos preditores de discordância entre RFR e FFR, é razoável que não sejam observados exatamente os mesmos preditores.^12,13^ Nossos achados mostram resultados semelhantes aos já relatados por Goto et al.^12^ e Kato et al.^13^ em relação à doença renal crônica como fator de risco para FP. Também encontramos síndrome coronariana aguda como fator de risco para FN e histórico de cardiopatia isquêmica crônica como fator protetor contra FP e não encontramos doença arterial periférica, sexo ou dimensões corporais, avaliadas como área de superfície corporal ou como índice de massa corporal, em nosso caso, como preditores de discordância, como foi o caso em estudos anteriores.

No entanto, um dos achados mais marcantes de ambos os estudos anteriores foi o de que as lesões da artéria descendente anterior esquerda comportaram-se como fator de risco para FP.^12,13^ Especificamente, o estudo de Kato et al.^13^ verificou que lesões que não acometiam a artéria descendente anterior esquerda se comportaram como fator de risco para FN, de forma semelhante aos nossos resultados. No presente estudo, no caso de variáveis complementares (por exemplo, presença ou ausência de doença renal crônica), optamos por avaliar o comportamento da variável menos comum como preditor de discordância. Considerando essa abordagem quanto à localização das lesões coronarianas (envolvimento versus não envolvimento da artéria descendente anterior esquerda), verificamos que, em pesquisas anteriores,^12,13^ a maioria das estenoses avaliadas envolvia a artéria descendente anterior esquerda, seguindo a prática padrão.^11,15–17^ Portanto, hipotetizamos que a condição de fator de risco para FP ou FN de acordo com a localização das estenoses coronarianas apresentaria aspectos complementares e, como a maioria das lesões corresponde à artéria descendente anterior esquerda, pareceu-nos mais adequado avaliar, como preditor de discordância, a de menor prevalência na prática padrão que, para lesões coronárias, é o envolvimento de outros territórios que não a artéria descendente anterior esquerda.

### Geração da “RFR Ajustada”: Por que ajustar para fatores de discordância?

Até o momento, estudos sobre índices da fisiologia coronária invasivos não consideraram integrar as informações fornecidas por parâmetros clínicos, e nosso trabalho é o primeiro a tentar isso. No entanto, o desenvolvimento de índices clínico-fisiológicos apresenta a questão de quais parâmetros incluir para reforçar os resultados dos índices coronarianos. Visto que os preditores de discordância são aqueles que contêm informações sobre as características específicas dos FPs e FNs, optamos por incluir apenas esses parâmetros em um índice global que possibilitaria reduzir erros na classificação diagnóstica dos pacientes.

No algoritmo proposto, as informações fornecidas pelos preditores independentes de discordância, juntamente com a RFR, foram integradas por meio de um modelo de regressão. O modelo subsequentemente atribuiu uma constante para o algoritmo e os coeficientes com seu sinal correspondente (positivo ou negativo) para cada variável. Por fim, o modelo foi avaliado, estabelecendo como limiar de corte otimizado um valor de “RFR Ajustada” ≤ 0,8172 para detectar valores de FFR ≤ 0,80.

Com base no exposto, o algoritmo deve ser interpretado da maneira seguinte. Para a “RFR Ajustada”, a variável com maior peso é a RFR, pois possui o maior coeficiente (+0,912), que pode ser modificado de forma incremental ou decrescente dependendo se o paciente possui algum ou todos os preditores de discordância. A presença de doença renal crônica (fator de risco para FP) aumenta o valor final da “RFR Ajustada”, facilitando a reclassificação do paciente como negativo, enquanto cardiopatia isquêmica crônica prévia (fator de proteção contra FP) o diminui, dificultando reclassificar o paciente negativo. De modo semelhante, lesões em territórios diferentes da artéria descendente anterior esquerda e síndrome coronariana aguda (ambos fatores de risco para FN) reduzem o valor final da “RFR Ajustada”, facilitando a reclassificação do paciente como positivo. Além disso, o algoritmo orienta não apenas a direção na qual reclassificar os pacientes, mas também de ponderar a influência dos preditores, de acordo com o peso de seus coeficientes.

Utilidade da “RFR Ajustada”: O desenvolvimento de índices clínico-fisiológicos pode ser clinicamente relevante?

A integração do resultado de um teste com as características clínicas do paciente é comum em diverso ambientes. Como exemplo, a estimativa mais precisa da função renal é obtida pela combinação dos valores de creatinina sérica com outros parâmetros como idade, peso e sexo.^21^ A “RFR Ajustada” permite uma capacidade diagnóstica aprimorada em comparação com o uso da RFR sozinha. Embora essa melhora tenha sido limitada em nossa população, os resultados sugerem que um índice clínico-fisiológico melhora todos os parâmetros diagnósticos. Isto é particularmente visível na melhoria das LRs, onde verificamos que a “RFR Ajustada” permite melhorar a utilidade do teste em comparação com a RFR na “zona cinzenta”.

### Limitações

Primeiro, o estudo de Casanova-Sandoval et al.^11^ foi um estudo realizado em um único país (Espanha), o que pode limitar sua extrapolação para outras populações. No entanto, sua natureza multicêntrica atenua essa limitação. Segundo, os critérios de inclusão do de Casanova-Sandoval et al.^11^ também permitiram o recrutamento de pacientes com síndrome coronariana aguda, apesar de a avaliação invasiva de lesões coronarianas ser recomendada principalmente em pacientes com angina estável. No entanto, na prática padrão, os índices coronarianos também são utilizados na síndrome coronariana aguda, o que tem sido apoiado na literatura^22^ e esse cenário também pode influenciar seus resultados. Terceiro, o tamanho limitado da amostra de nosso estudo pode ser estendido e a metodologia usada pode ser modificada em estudos subsequentes para refinar ainda mais a construção de índices combinados. No entanto, nossa pesquisa encontrou uma melhora em todos os parâmetros diagnósticos. Por fim, cabe destacar que, além de estudos que permitam a derivação de novos índices clínico-fisiológicos, são necessários estudos de validação em populações externas.

## Conclusões

Ajustar a RFR integrando as informações fornecidas pelos preditores de discordância para obter a “RFR Ajustada” melhorou a capacidade diagnóstica em nossa população. O desenvolvimento de índices clínico-fisiológicos, incluindo RFR ou outros índices, poderia melhorar a capacidade diagnóstica dos índices da fisiologia coronária. Estudos futuros em grandes populações são necessários para avaliar a utilidade de metodologias semelhantes para refinar estudos da fisiologia coronária.

### O que já se sabe sobre o assunto?

Alterações mínimas nos limiares de corte dos testes da fisiologia coronária levam a mudanças significativas na sensibilidade, especificidade e valores preditivos. Além disso, existe variabilidade entre os limiares de corte da RFR nas diferentes séries, sugerindo uma influência das características da população na capacidade diagnóstica desse índice. Já foram documentados preditores de discordância entre os resultados fornecidos pela RFR e o diagnóstico “padrão ouro” para testes da fisiologia coronária, a saber, a FFR. Esses preditores parecem úteis para complementar as informações oferecidas pela RFR na “zona cinzenta” de valores.

### O que há de novo?

Doença renal crônica, envolvimento de outras artérias que não a descendente anterior esquerda, indicação de síndrome coronariana aguda e histórico de cardiopatia isquêmica crônica têm se mostrado preditores independentes de discordância na “zona cinzenta” da RFR em comparação com a FFR. A construção de um índice clínico-fisiológico modificado (a “RFR Ajustada”) que inclui informações sobre a RFR e preditores de discordância, melhorou a capacidade diagnóstica na “zona cinzenta”. O desenvolvimento de índices clínico-fisiológicos pode ser útil para melhorar tanto a capacidade diagnóstica da RFR quanto de outros índices da fisiologia coronária.

## *Material suplementar

Para informação adicional, por favor, clique aqui.
