# Supplementary material for: Ajustando a RFR por Preditores de Discordância, “A RFR Ajustada”: Uma Metodologia Alternativa para Melhorar a Capacidade Diagnóstica dos Índices Coronarianos
Source: Arq Bras Cardiol. 2022 Nov 1;119(5):705–13. [Article in Portuguese] doi: 10.36660/abc.20220176 (PMC9750217; doi:10.36660/abc.20220176)
Supplement: Translation

# Translation: Adjusting RFR by Predictors of Disagreement, “The Adjusted RFR”: An Alternative Methodology to Improve the Diagnostic Capacity of Coronary Indices

**Authors:** Diego Fernández-Rodríguez, Juan Casanova-Sandoval, Ignacio Barriuso, Kristian Rivera, Imanol Otaegui, Bruno García del Blanco, Teresa Gil Jiménez, Manuel López-Pérez, Marcos Rodríguez-Esteban, Francisco Torres-Saura, Víctor Jiménez Díaz, Raymundo Ocaranza-Sánchez, Vicente Peral Disdier, Guillermo Sánchez Elvira, Fernando Worner

**Affiliations:** 1 Lleida Spain Hospital Universitari Arnau de Vilanova, Lleida – Spain; 2 Lleida Spain Institut de Recerca Biomédica de Lleida (IRBLleida), Lleida – Spain; 3 Barcelona Spain Hospital Universitari Vall d´Hebron, Barcelona – Spain; 4 Granada Spain Hospital Universitario Clínico San Cecilio, Granada – Spain; 5 Tenerife Spain Hospital Universitario Nuestra Señora de Candelaria, Tenerife – Spain; 6 Elche Spain Hospital Universitario de Vinalopó, Elche – Spain; 7 Vigo Spain Hospital Universitario Alvaro Cunqueiro, Vigo – Spain; 8 Lugo Spain Hospital Universitario Lucus Augusti, Lugo – Spain; 9 Palma de Mallorca Spain Hospital Son Espases, Palma de Mallorca – Spain; 10 Pamplona Spain Complejo Hospitalario de Navarra, Pamplona – Spain

**Keywords:** Angina, Fractional Flow Reserve, Resting Full-cycle Ratio, Sensitivity, Specificity

## Abstract

**Background:**

Cutoff thresholds for the “resting full-cycle ratio” (RFR) oscillate in different series, suggesting that population characteristics may influence them. Likewise, predictors of discordance between the RFR and fractional flow reserve (FFR) have been documented. The RECOPA Study showed that diagnostic capacity is reduced in the RFR “grey zone”, requiring the performance of FFR to rule out or confirm ischemia.

**Objectives:**

To determine predictors of discordance, integrate the information they provide in a clinical-physiological index, the “Adjusted RFR”, and compare its agreement with the FFR.

**Methods:**

Using data from the RECOPA Study, predictors of discordance with respect to FFR were determined in the RFR “grey zone” (0.86 to 0.92) to construct an index (“Adjusted RFR”) that would weigh RFR together with predictors of discordance and evaluate its agreement with FFR.

**Results:**

A total of 156 lesions were evaluated in 141 patients. Predictors of discordance were: chronic kidney disease, previous ischemic heart disease, lesions not involving the anterior descending artery, and acute coronary syndrome. Though limited, the “Adjusted RFR” improved the diagnostic capacity compared to the RFR in the “grey zone” (AUC-RFR = 0.651 versus AUC-“Adjusted RFR” = 0.749), also showing an improvement in all diagnostic indices when optimal cutoff thresholds were established (sensitivity: 59% to 68%; specificity: 62% to 75%; diagnostic accuracy: 60% to 71%; positive likelihood ratio: 1.51 to 2.34; negative likelihood ratio: 0.64 to 0.37).

**Conclusions:**

Adjusting the RFR by integrating the information provided by predictors of discordance to obtain the “Adjusted RFR” improved the diagnostic capacity in our population. Further studies are required to evaluate whether clinical-physiological indices improve the diagnostic capacity of RFR or other coronary indices.

## Introduction

Coronary physiological indices are an essential tool in decision making in ischemic heart disease.^1,2^ In clinical practice, they are used dichotomously to determine the functional significance of coronary lesions.^3,4^ However, the choice of cutoff thresholds (CoTs) using receiver operating characteristic (ROC) curve analysis means that minimal changes in CoT may lead to relevant changes in sensitivity and specificity.^5–7^ Furthermore, the optimal CoTs vary between series, suggesting that the heterogeneity of the study populations may influence the diagnostic capacity of these indices.^5–7^

The concept of the “grey zone” in the coronary flow reserve or fractional flow reserve (FFR)^8,9^ refers to a range of values close to the CoT whose extremes have high predictive values to confirm or rule out ischemia. This concept has also been studied with non-hyperemic resting indices (NHRIs).^10,11^ The RECOPA Study^11^ was a validation study of the “resting full-cycle ratio” (RFR) against FFR in “real life” which also evaluated the usefulness of a hybrid strategy of RFR and FFR for the functional assessment of “grey zone” stenosis.^11^

On the other hand, there is growing interest in determining predictors of discordance between NHRIs and FFR. Recent studies^12,13^ have identified some of them for RFR. However, to the best of our knowledge, the information from these predictors has not been used to improve the diagnostic capacity of coronary indices in general, or of NHRIs in particular.

Therefore, the objective of this study was to determine predictors of discordance between RFR and FFR and to integrate this information to construct a modified index of RFR, the “Adjusted RFR”, which allows for improving the diagnostic capacity with respect to RFR in the “grey zone”.

## Material and methods

### Study population

The population of this study was selected using data from the RECOPA Study, the details and results of which have previously been published.^11^ This study was approved by the Ethics Committee of each site, meeting the requirements and standards of the Declaration of Helsinki and its subsequent amendments, as well as applicable data protection regulations.

To summarize, the RECOPA Study^11^ was a validation study of RFR versus FFR in standard practice, where 380 coronary lesions in 311 patients were functionally evaluated by pressure guidance, obtaining RFR and FFR values. The thresholds for detecting ischemia were RFR ≤ 0.89 and FFR ≤ 0.80, with correlation levels (R^2^ = 0.81; p < 0.001), sensitivity (76%) and specificity (80%), similar to those reported by other “real life” studies. However, their application to the study population showed limited predictive values (positive predictive value [PPV] = 68%; negative predictive value [NPV] = 80%). Therefore, a “grey zone” (RFR from 0.86 to 0.92) was determined in which to assess the functional impact of stenoses using both techniques (hybrid RFR-FFR strategy), making it possible to obtain high predictive values (PPV = 91%; NPV = 92%) and to reduce the administration of vasodilators by 58%.

Since the extreme values of the RFR make it possible to obtain a very high agreement, concentrating the discrepancy between both techniques in the “grey zone”, only lesions with RFR of 0.86 to 0.92 were selected, ultimately including a total of 156 lesions, corresponding to 141 patients.

Determination of the Predictors of Discordance and Establishment of the “Adjusted RFR”

RFR^14^ is an NHRI that evaluates the hemodynamic significance of coronary stenoses, identifying the minimum ratio between blood pressure distal to the coronary stenosis (Pd) and aortic blood pressure (Pa) throughout the cardiac cycle. A CoT of RFR ≤ 0.89 is considered the most adequate for determining the presence of ischemia, despite variations in the optimal CoTs reported in the different series.^11–17^ In an attempt to fine-tune the agreement with the FFR in the “grey zone” (RFR from 0.86 to 0.92).^11^ an analysis was performed to determine the predictors of discordance between both techniques, and information provided by them was then included in the construction of a new index: “Adjusted RFR”.

First, lesions were grouped into 4 groups according to the functional study result: RFR-/FFR- (true negative), RFR+/FFR- (false positive [FP]), RFR-/FFR+ (false negative [FN]), and RFR+/FFR+ (true positive), comparing the clinical and angiographic characteristics between each group. Subsequently, the groups with discordant results were selected: RFR+/FFR- (FP) and RFR-/FFR+ (FN); independent predictors of discordance were then determined for each group. Finally, the “Adjusted RFR” was constructed including the RFR and the predictors of discordance, assigning them their corresponding weighting coefficients.

### Statistical analysis

The statistical analyses were performed using SPSS version 20.0 software (IBM Corp., Armonk, NY, USA), with two-tailed values of p < 0.05 considered statistically significant. Categorical variables were presented as numbers and relative frequencies (percentages), and continuous variables as mean (standard deviation) or median with range or interquartile range depending on their distribution. Continuous variables were compared using Student’s t test for unpaired samples, and categorical variables were compared using the chi-squared test or Fisher’s exact test, as appropriate. Mann–Whitney U tests were used for non-parametric data.

To identify predictors of discordance, for both FP and FN, binary logistic regression models were used, including in the final multivariate analysis those predictors with values ≤ 0.10 in the univariate analysis. Results were given as odds ratio (OR) with 95% confidence interval (95% CI). Once the predictors of discordance were obtained, the “Adjusted RFR” was constructed using linear regression to establish a predictive model of FFR that contemplates the value of the RFR and the predictors of discordance, assigning them a coefficient that weighed their relevance using the following algorithm:


“Adjusted RFR”: $$\text{p}(\text{y}=\text{FFR})=\text{RFR Adjusted}={\text{β}}_{\text{cte}}+{\text{β}}_{\text{RFR}}*\text{RFR}+\ldots \ldots +{\text{β}}_{\text{n}}*{\text{X}}_{\text{n}}$$

*Weighted coefficients (β_i_) could be positive or negative depending on whether predictors were protective or risk factors for being FN or FP.

Finally, sensitivity and specificity analyses were performed, also estimating the optimal CoT of the “Adjusted RFR” to obtain an FFR value ≤ 0.80, using ROC curve analysis. Positive and negative likelihood ratios (LR+ and LR-) were also calculated for RFR and “Adjusted RFR”, considering the test utility as follows:^14^

– LR+: < 2 (not useful); 2 to 5 (fair); 5 to 10 (good); > 10 (excellent).
– LR−: > 0.5 (not useful); 0.5 to 0.2 (fair); 0.2 to 0.1 (good); < 0.1 (excellent).

## Results

In this study, 141 patients, with a total of 156 lesions, were included. A single lesion was explored in most patients, with 4 being the maximum number of lesions evaluated in 1 patient.

### Clinical and angiographic characteristics

Baseline characteristics per patient are shown in Table 1. Table 2 shows, per lesion, the baseline characteristics of the 4 comparison groups, observing that FPs (RFR+/FFR-) were older, and they had a greater prevalence of chronic kidney disease and a greater percentage of previous chronic ischemic heart disease. As regards FNs (RFR-/FFR+), a greater prevalence of active smoking and acute coronary syndrome was found. Table 3 also shows, per lesion, the angiographic and physiological characteristics of the comparison groups, noting that FNs (RFR-/FFR+) had a higher percentage of lesions not involving the left anterior descending artery compared to all other groups. The specifically affected coronary segment is presented in the Supplementary Material. In addition, a gradient in RFR and Pd/Pa values was observed between the 4 comparison arms.

**Table 1:** Baseline characteristics per patient

		Patients (n=141)
**Age**, *(years), mean (SD)*		65.82 (12.3)
**Female sex**, *n (%)*		39 (27.7%)
**BMI**, *(kg/m^2^), mean (SD)*		28.0 (4.8%)
**Hypertension**, *n (%)*		104 (73.8%)
**Dyslipidemia**, *n (%)*		93 (66%)
**Diabetes mellitus**, *n (%)*		50 (35.5%)
**Current smoker**, *n (%)*		26 (18.4%)
**Previous chronic ischemic heart disease**, *n (%)*		40 (28.4%)
**Cerebrovascular disease**, *n (%)*		13 (9.2%)
**Atrial fibrillation**, *n (%)*		15 (10.6%)
**Peripheral vascular disease, n (%)**		13 (9.2%)
**COPD**, *n (%)*		9 (6.4%)
**Glomerular filtration rate**, *(mL/min/1.73 m*^2^*), mean (SD)*		74.0 (31.2)
**Chronic kidney disease**, *(glomerular filtration rate < 60 ml/min/1.73 m*^2^), *n (%)*		51 (36.7%)
**Clinical indication**, *n (%)*		
	– Stable angina	102 (72.3%)
	– Non ST-segment elevation ACS: culprit lesion	21 (14.9%)
	– Non ST-segment elevation ACS: non-culprit lesion	10 (7.1%)
	– ST-segment elevation ACS: non-culprit lesion	8 (5.7%)
**Lesions/patient**, *(n), median (minimum-maximum)*		1 (1-4)

ACS: acute coronary syndrome; BMI: body mass index; COPD: chronic obstructive pulmonary disease; SD: standard deviation.

**Table 2:** Baseline characteristics per lesion

		TN: RFR-/FFR- (n=60)	FP: RFR+/FFR- (n=41)	FN: RFR-/FFR+ (n=21)	TP: RFR+/FFR+ (n=34)	p value
**Age**, *(years), mean (SD)*		63.0 (14.0)	71.5 (10.3)	62.1 (8.7)	66.2 (10.9)	**0.002**
**Female sex**, *n (%)*		18 (30.0%)	13 (31.7%)	5 (23.8%)	4 (11.8%)	0.182
**BMI**, *(Kg/m*^2^*), mean (SD)*		27.8 (4.6)	27.7 (4.8)	29.2 (5.5)	27.8 (4.2)	0.647
**Hypertension**, *n (%)*		43 (71.7%)	30 (73.2%)	18 (85.7%)	24 (70.6%)	0.600
**Dyslipidemia**, *n (%)*		38 (63.3%)	25 (61.0%)	13 (61.9%)	22 (64.7%)	0.989
**Diabetes mellitus**, *n (%)*		20 (33.3%)	18 (43.9%)	4 (19.0%)	14 (41.2%)	0.229
**Current smoker**, *n (%)*		14 (23.3%)	3 (7.3%)	9 (42.9%)	7 (20.6%)	**0.013**
**Previous chronic ischemic heart disease**, *n (%)*		26 (43.3%)	5 (12.2%)	8 (38.1%)	7 (20.6%)	**0.004**
**Cerebrovascular disease**, *n (%)*		5 (8.3%)	5 (12.2%)	2 (9.5%)	2 (5.9%)	0.862
**Atrial fibrillation**, *n (%)*		4 (6.7%)	6 (14.6%)	1 (4.8%)	4 (11.8%)	0.468
**Peripheral vascular disease**, *n (%)*		5 (8.3%)	4 (9.8%)	0 (0%)	6 (17.6%)	0.181
**COPD**, *n (%)*		6 (10.0%)	0 (0%)	0 (0%)	4 (11.8%)	0.061
**Glomerular filtration rate**, *(mL/min/1.73 m*^2^), *mean (SD)*		78.4 (33.0)	57.5 (26.0)	90.1 (20.4)	74.4 (29.5)	**< 0.001**
**Chronic kidney disease**, *(glomerular filtration rate < 60 ml/min/1.73 m*^2^), *n (%)*		19 (31.7%)	26 (63.4%)	2 (9.5%)	9 (26.5%)	**< 0.001**
**Clinical indication**, *n (%)*						**0.012**
	- *Stable angina*	46 (76.7%)	33 (80.5%)	9 (42.9%)	24 (70.6%)	
	- *Acute coronary syndrome*	14 (23.3%)	8 (19.5%)	12 (57.1%)	10 (29.4%)	

BMI: body mass index; COPD: chronic obstructive pulmonary disease; FFR: fractional flow reserve; FN: false negative; FP: false positive; RFR: resting full-cycle ratio; SD: standard deviation; TN: true negative; TP: true positive.

**Table 3:** Angiographic and physiological characteristics per lesion

		TN: RFR-/FFR- (n=60)	FP: RFR+/FFR- (n=41)	FN: RFR-/FFR+ (n=21)	TP: RFR+/FFR+ (n=34)	p value
**Adenosine administration**, *n (%)*						0.343
	- *Adenosine intravenous*	18 (30.0%)	17 (51.5%)	10 (47.6%)	10 (29.4%)	
	- *Adenosine intracoronary*	42 (70.0%)	24 (58.5%)	11 (52.4%)	24 (70.6%)	
**Guide catheter size**, *n (%)*						0.574
	- *5 French*	2 (3.3%)	2 (4.9%)	0 (0%)	1 82.9%)	
	- *6 French*	58 (96.7%)	39 (95.1%)	21 (100%)	32 (94.1%)	
	- *7 French*	0 (0%)	0 (0%)	0 (0%)	1 (2.9%)	
**Affected vessel**, *n (%)*						**0.019**
	- *LAD*	43 (71.7%)	33 (80.5%)	11 (52.4%)	30 (88.2%)	
	- *Non-LAD*	17 (28.3%)	8 (19.5%)	10 (47.6%)	4 (11.8%)	
**Percentage of stenosis**, *(%), mean (SD)*		57 (11)	57 (10)	61 (10)	61 (9)	0.136
**Length of the lesion**, *n (%)*						0.716
	- *<12 mm*	30 (50.0%)	17 (41.5%)	11 (52.4%)	13 (38.2%)	
	- *12 to 25 mm*	23 (38.3%)	20 (48.8%)	9 (42.9%)	14 (44.1%)	
	- *>25 mm*	7 (11.7%)	4 (9.8%)	1 (4.8%)	6 (17.6%)	
**Vessel diameter**, *(mm), mean (SD)*		3.01 (0.53)	2.89 (0.37)	2.93 (0.53)	2.92 (0.45)	0.636
**Coronary indices**, *mean (SD)*						
	- *RFR*	0.91 (0.01)	0.88 (0.01)	0.91 (0.01)	0.87 (0.01)	**<0.001**
	- *Pd/Pa*	0.93 (0.02)	0.92 (0.02)	0.92 (0.02)	0.90 (0.03)	**<0.001**
	- *FFR*	0.86 (0.03)	0.85 (0.03)	0.76 (0.04)	0.76 (0.03)	**<0.001**

FFR: “fractional flow reserve”; FN: false negative; FP: false positive; LAD: affectation of the left anterior descending artery; non-LAD: not affecting the left anterior descending artery; Pd/Pa: ratio distal coronary pressure/aortic pressure; RFR: “resting full-cycle ratio”; SD: standard deviation; TN: true negative; TP: true positive.

### Determination of predictors of discordance

Table 4 shows the independent predictors of discordance for FP and FN. As regards FP (RFR+/FFR-), chronic kidney disease was identified as an independent risk factor for discordance (OR 3.224; 1.386 to 7.501; p = 0.007). In contrast, a history of chronic ischemic heart disease was shown to be a protective factor against discordance (OR 0.296; 0.102 to 0.858; p = 0.025). As regards FN (RFR-/FFR+), the clinical context of acute coronary syndrome (OR 3.687; 1.247 to 10.899; p = 0.018) and lesions in a location other than the left anterior descending artery (OR 3.529; 1.231 to 10.118; p = 0.019) were finally identified as independent risk factors for discordance.

**Table 4:** Independent predictors of discordance

RFR+/FFR- (false positives)							
Univariate analysis	OR	CI (95%)	P value	Multivariate analysis	OR	CI (95%)	p value
**CKD**	**4.911**	**2.298-10.498**	**<0.001**	**DRC**	**3.224**	**1.386-7.501**	**0.007**
**Age ≥ 75 years**	**3.981**	**1.862-8.511**	**<0.001**	**CIC prévia**	**0.296**	**0.102-0.858**	**0.025**
**Non-LAD lesions**	0.657	0.274-1.576	0.345				
**ACS**	0.532	0.224-1.266	0.150				
**Previous CIHD**	**0.251**	**0.091-0.688**	**0.005**				
**Current smoking**	**0.224**	**0.064-0.688**	**0.005**				
**RFR-/FFR+ (false negatives)**							
**Univariate analysis**	**OR**	**CI (95%)**	**P value**	**Multivariate analysis**	**OR**	**CI (95%)**	**p value**
**ACS**	**4.292**	**1.658-11.107**	**0.002**	**SCA**	**3.687**	**1.247-10.899**	**0.018**
**Current smoking**	**3.469**	**1.314-9.154**	**0.009**	**Lesões não DAE**	**3.529**	**1.231-10.118**	**0.019**
**Non-LAD lesions**	**3.323**	**1.285-8.594**	**0.010**				
**Previous CIHD**	1.157	0.603-4.091	0.352				
**CKD**	**0.158**	**0.035-0.706**	**0.003**				
**Age ≥ 75 years**	**0.103**	**0.013-0.796**	**0.003**				

ACS: acute coronary syndrome; CI: confidence interval; CIHD: chronic ischemic heart disease; CKD: chronic kidney disease; FFR: “fractional flow reserve”; non-LAD lesions: lesions not involving the left anterior descending artery; OR: odds ratio; RFR: “resting full-cycle ratio”.

### Generation of the “Adjusted RFR”

Finally, the RFR value and independent predictors were included in the model to generate the “Adjusted RFR”. The algorithm with the coefficients corresponding to each predictor is shown below:


“Adjusted RFR”: $$\begin{array}{l} \text{Adjusted RFR}=0.009+0.912\text{*RFR}+0.023\text{*CKD}-0.019\text{*non-LAD}\\ -0.017\text{*ACS}-0.005\text{*previous CIHD} \end{array}$$

Abbreviations: RFR: “resting full-cycle ratio”; CKD: chronic kidney disease (glomerular filtration rate < 60 ml/min/1.73 m^2^); non-LAD: lesions not affecting the left anterior descending artery; ACS: acute coronary syndrome; previous CIHD: history of chronic ischemic heart disease.

This algorithm shows that chronic kidney disease (risk factor for FP) is entered with a positive sign. Lesions not affecting the left anterior descending artery and indication for acute coronary syndrome (both risk factors for FN), as well as history of chronic ischemic heart disease (protective factor for FP), are entered with a negative sign.

### Sensitivity and specificity analysis

Figure 1 shows the comparative ROC curves for RFR and the “Adjusted RFR”. An increase was seen in the area under the curve (AUC) of the “Adjusted RFR” with respect to the RFR, from 0.651 to 0.749, determining as optimal CoT an “Adjusted RFR” of ≤ 0.8172 to detect FFR values ≤ 0.80. Likewise, Figure 2 compares the contingency tables of both indices according to the established CoTs, with improvements in sensitivity ranging from 59% to 68%, specificity ranging from 62% to 75%, diagnostic accuracy ranging from 60% to 71%, PPV ranging from 45% to 56%, and NPV ranging from 74% to 83%. Of particular interest is the improvement in LRs, where we found that, with the new index, LR+ increased from 1.51 to 2.34 and LR- decreased from 0.64 to 0.37. Thus, the “Adjusted RFR” has satisfactory utility compared to RFR, which is not useful for discriminating patients in the “grey zone”.

**Figure 1:** ROC curves of RFR versus FFR ≤ 0.80 and “Adjusted RFR” and FFR ≤ 0.80. AUC: area under the curve; FFR: fractional flow reserve; ROC: receiver operating characteristic; RFR: resting full-cycle ratio. The ROC curve showed an AUC for the RFR of 0.651 (0.559 to 0.744; p = 0.002), improving the AUC for “Adjusted RFR” to 0.749 (0.669 to 0.828; p < 0.001) and establishing as optimum cutoff threshold for “Adjusted RFR” a value of 0.8172.

**Figure 2:** Comparison of diagnostic parameters according to 2 × 2 Tables for the cutoff thresholds of RFR (≤ 0.89) and “Adjusted RFR” (≤ 0.8172) versus FFR (≤ 0.80). FFR: fractional flow reserve; FN: false negative; FP: false positive; NPV: negative predictive value; LR+: positive likelihood ratio; LR-: negative likelihood ratio; PPV: positive predictive value; RFR: resting full-cycle ratio; TN: true negative; TP: true positive.

## Discussion

The main findings of the study were: a) chronic kidney disease, involvement of arteries other than the left anterior descending artery, indication for acute coronary syndrome, and history of chronic ischemic heart disease were shown as independent predictors of discordance in the “grey zone” of the RFR with respect to FFR; and b) the modification of the RFR by including independent predictors of discordance (“Adjusted RFR”) made it possible to improve the diagnostic capacity of the test for “grey zone” values.

### Selection of the target population: Why the “grey zone”?

Any continuous quantitative index used dichotomously involves some degree of diagnostic uncertainty in values close to the established CoT.^18^ The concept of “grey zone” for coronary physiological indices arises from validation studies of the FFR for the detection of ischemia induced by epicardial coronary stenosis.^8,9,19,20^ This concept was subsequently extended to other NHRIs such as the instantaneous wave-free ratio and the RFR, showing that extreme NHRI values showed very high agreement with FFR, and, in values close to CoT (“grey zone”), the diagnostic capacity decreased.^10,11^ Since it is plausible that the few discordant results between RFR and FFR in case of extreme RFR values are mainly related to errors in the measurement technique, it was decided to restrict the determination of predictors of discordance to the “grey zone” of RFR.

In addition, the proportion of patients assessed by invasive physiological study located in the “grey zone” is relevant, showing in data on RFR and instantaneous wave-free ratio that the proportion of patients may exceed 40%.^10,11^ Therefore, we consider it essential to develop diagnostic tools that make it possible to refine the diagnosis of NHRI and eventually other invasive physiological indices, of either epicardial circulation or coronary microcirculation, for this range of values.

Assessment of predictors of discordance: Does the affected coronary territory predict false positives or false negatives?

Given the limited sample size and the heterogeneity of the study populations assessed by the predictors of discordance between RFR and FFR, it is reasonable that exactly the same predictors are not observed.^12,13^ Our findings show results similar to those already reported by Goto^12^ and Kato^13^ regarding chronic kidney disease as a risk factor for FP. We also found acute coronary syndrome to be a risk factor for FN and a history of chronic ischemic heart disease to be a protective factor for FP, and we did not find peripheral arterial disease, sex, or body dimensions, assessed as body surface area or as body mass index in our case, to be predictors of discordance as in previous studies.

However, one of the most striking findings of both previous studies was that left anterior descending artery lesions behaved as a risk factor for FP.^12,13^ Specifically, Kato’s study^13^ found that lesions not affecting the left anterior descending artery behaved as a risk factor for FN, in a similar way to our own results. In this study, in the case of complementary variables (e.g., presence or absence of chronic kidney disease), we decided to assess the behavior of the least common variable as a predictor of discordance. Considering this approach regarding the location of coronary lesions (involvement versus non-involvement of the left anterior descending artery), we found that, in previous research,^12,13^ the majority of evaluated stenoses involved the left anterior descending artery, in line with standard practice.^11,15–17^ Thus, we hypothesized that the condition of risk factor for FP or FN according to the location of coronary stenoses would show complementary aspects, and since most lesions correspond to the left anterior descending artery, it seemed more appropriate to assess, as a predictor of discordance, that of lower prevalence in standard practice, which for coronary lesions is the involvement of territories other than the left anterior descending artery.

### Generation of the “Adjusted RFR”: Why adjust for discordance factors?

To date, studies on invasive coronary physiological indices have not considered integrating the information provided by clinical parameters, and *our work is the first to attempt this.* However, the development of clinical-physiological indices presents the question of which parameters to include to reinforce the results of coronary indices. Since the predictors of discordance are those that contain information about the specific characteristics of FPs and FNs, we chose to only include these parameters in a global index that would make it possible to reduce errors in the diagnostic classification of patients.

In the proposed algorithm, the information provided by the independent predictors of discordance, together with the RFR, was integrated using a regression model. The model subsequently assigned a constant for the algorithm and the coefficients with their corresponding sign (positive or negative) for each variable. Finally, the model was evaluated, establishing as optimal CoT a value of “Adjusted RFR” ≤ 0.8172 to detect FFR values ≤ 0.80.

Based on the above, the algorithm should be interpreted as follows. For “Adjusted RFR”, the variable with the greatest weight is RFR since it has the highest coefficient (+0.912), which may be modified incrementally or decrementally depending on whether the patient has any or all predictors of discordance. The presence of chronic kidney disease (risk factor for FP) increases the final value of the “Adjusted RFR”, making it easier to reclassify the patient as negative, while previous chronic ischemic heart disease (FP protective factor) decreases it, making it difficult to reclassify the negative patient. Similarly, lesions in territories other than the left anterior descending artery and acute coronary syndrome (both risk factors for FN) reduce the final value of the “Adjusted RFR”, making it easier to reclassify the patient as positive. In addition, the algorithm guides not only the direction in which to reclassify patients but also to weigh the influence of the predictors, according to the weight of their coefficients.

Utility of the “Adjusted RFR”: Can the development of clinical-physiological indices be clinically relevant?

The integration of the result of a test with the clinical characteristics of the patient is common in multiple settings. As an example, the most precise estimate of renal function is obtained by combining serum creatinine values with other parameters such as age, weight, and sex.^21^ The “Adjusted RFR” allows for an improved diagnostic capacity compared to the use of RFR alone. Although such improvement was limited in our population, the results suggest that a clinical-physiological index improves all diagnostic parameters. This is particularly visible in the improvement of LRs, where we found that “Adjusted RFR” allows for improvement of test utility compared to the RFR in the “grey zone”.

### Limitations

First, the RECOPA Study^11^ was a single-country study (Spain), which may limit its extrapolation to other populations. However, its multicenter nature attenuates this limitation. Second, the inclusion criteria of the RECOPA Study^11^ also allowed the recruitment of patients with acute coronary syndrome, despite the fact that invasive assessment of coronary lesions is mainly recommended in patients with stable angina. However, in standard practice, coronary indices are also used in acute coronary syndrome, which has been supported in the literature,^22^ and this scenario may also influence its results. Third, the limited sample size of our study could be extended, and the methodology used could be modified in subsequent studies to further refine the construction of combined indices. However, our research found an improvement in all diagnostic parameters. Finally, it should be noted that, in addition to studies allowing for the derivation of new clinical-physiological indices, validation studies are required in external populations.

## Conclusions

Adjusting the RFR by integrating the information provided by the predictors of discordance to obtain the “Adjusted RFR” improved the diagnostic capacity in our population. The development of clinical-physiological indices, including RFR or other indices, could improve the diagnostic capacity of coronary physiological indices. Future studies in large populations are required to assess the utility of similar methodologies in refining coronary physiology studies.

### What is known about the topic?

Minimal changes in CoTs of coronary physiology tests lead to significant changes in sensitivity, specificity, and predictive values. In addition, variability exists between the CoTs of RFR in the different series, suggesting an influence of population characteristics on the diagnostic capacity of this index. Predictors of discordance have already been documented between the results offered by the RFR and the diagnostic “gold standard” for coronary physiology tests, namely, the FFR. These predictors seem useful to complement the information offered by the RFR for values in the “grey zone”.

### What’s new?

Chronic kidney disease, involvement of arteries other than the left anterior descending artery, indication for acute coronary syndrome, and a history of chronic ischemic heart disease have been shown to be independent predictors of discordance in the “grey zone” of RFR as compared to FFR. The construction of a modified clinical-physiological index (the “Adjusted RFR”) that includes information on the RFR and predictors of discordance improved the diagnostic capacity in the “grey zone”. The development of clinical-physiological indices could be useful to improve both the diagnostic capacity of RFR and other coronary physiological indices.

## *Supplemental Materials

For additional information, please click here.
